# Technostress, technosuffering, and relational strain: a multi-method qualitative study of how remote and digital work affects staff in UK general practice

**DOI:** 10.3399/BJGP.2024.0322

**Published:** 2025-02-11

**Authors:** Francesca H Dakin, Nina Hemmings, Asli Kalin, Lucy Moore, Emma Ladds, Rebecca Payne, Rebecca Rosen, Richard Byng, Joseph Wherton, Sietse Wieringa, Trisha Greenhalgh

**Affiliations:** Nuffield Department of Primary Care Health Sciences, University of Oxford, Oxford.; Nuffield Trust, London.; Nuffield Department of Primary Care Health Sciences, University of Oxford, Oxford.; Nuffield Department of Primary Care Health Sciences, University of Oxford, Oxford.; Nuffield Department of Primary Care Health Sciences, University of Oxford, Oxford.; Nuffield Department of Primary Care Health Sciences, University of Oxford, Oxford.; Nuffield Trust, London.; Peninsula Schools of Medicine and Dentistry, University of Plymouth, Plymouth.; Nuffield Department of Primary Care Health Sciences, University of Oxford, Oxford.; Nuffield Department of Primary Care Health Sciences, University of Oxford, Oxford.; Nuffield Department of Primary Care Health Sciences, University of Oxford, Oxford.

**Keywords:** digitalisation, general practice, job satisfaction, technostress, workforce, workload

## Abstract

**Background:**

The introduction of remote and digital forms of working in UK general practice has driven the development of new routines and working styles.

**Aim:**

To explore and theorise how new forms of work have affected general practice staff.

**Design and setting:**

Multi-sited, qualitative case study in UK general practice.

**Method:**

Using longitudinal ethnography by researchers in residence, we followed 12 practices for 28 months (September 2021 to December 2023). This core dataset was supplemented by workshops and stakeholder interviews. Data analysis applied theories from the sociology of work, organisation studies, and internet studies.

**Results:**

Staff made significant efforts to adapt to and embed digital services into their work. When technologies work well they can offer improved convenience, efficiency, more comprehensive patient care, and workplace fulfilment for staff. However, for many clinical and administrative staff, compromises and frictions embedded in digitalised workplace routines and processes could also lead to job dissatisfaction, worsened wellbeing, and misalignments with professional values and identities. We found that this workplace suffering caused relational strain between team members and had an impact on team cohesiveness and coordination.

**Conclusion:**

The digitalisation of working routines in UK general practice poses a unique challenge to the workforce, risking technostress, workplace suffering, and increased relational strain within and between teams. To embed the benefits of digitalisation, we must first improve practice teams’ readiness for change, which includes strengthening practices’ relational structures that provide support during periods of adaptation. Practices must be empowered to determine a locally appropriate configuration of digital tools and given the resources and time to adapt working routines.

## Introduction

Since 2020, changes in the organisation and delivery of UK general practice have been extensive and far reaching. Some, such as total triage^1^ and remote-by-default consultations by telephone, video, or electronic communication,^2^ went from intermittent use to routine practice as part of an emergency infection control strategy, and subsequently retained (at least in part) as elements of a long-term policy vision for a more accessible and efficient service.^3^^–^7 These changes need to be interpreted in the context of chronic underfunding in the UK public sector in general,^8^ and general practice in particular,^9^ rising burden of demand,^10^ and task-shifting from other sectors under strain — for example, secondary care and social care^8^^,^^11^ — which creates challenges for implementing complex changes to working routines. The policy assumption has been that technology-enabled work such as remote booking and consulting can improve labour productivity, affording clinicians the ‘gift of time’,^3^ and improve convenience for patients, although studies of remote and digital access to UK general practice have not demonstrated clear efficiency gains.^12^

We and others have previously reported that the expansion of remote and digital modalities in UK general practice has generated safety concerns,^13^^–^17 unmet training needs,^17^^,^^18^ threats to continuity of care,^19^^,^^20^ challenges to coordination of care,^7^^,^^20^ widening inequities of access,^21^^–^23 and a range of workforce issues including reduced job satisfaction, increased job stress, loss of confidence, low morale, increased turnover of staff, and increased sickness absence.^17^^,^^18^^,^^24^^,^^25^

In the work reported here, we aimed to build a more nuanced understanding of the impact of digitalisation on the UK primary care workforce and produce a theoretical framework to better understand why many staff are currently so troubled and unfulfilled, and how this can have an impact on the resilience and effectiveness of the team and the organisation. We combine Ragu-Nathan *et al*’s concept of technostress^26^ with Gill’s concept of workplace suffering to theorise how technostress leads to suffering at work.^27^ We employ Dakin *et al*’s interpretation of the ‘relational workplace’^24^ to explore the strain that this suffering places on team relations, with adverse effects on mutual accountability and support.

**Table:** How this fits in

The digitalisation of UK general practice has had far-reaching implications on the daily working routines of clinical and support staff. Although technologies can offer improvements to work, their integration and ongoing use have caused unique forms of stress, suffering, and strain on individuals and teams. To reduce these negative effects, attention must be paid to practice teams’ readiness for change, and to strengthening the relational structures that enable better resilience to change. Practices should be enabled to determine locally what digital innovations will work best for them, allowing appropriate time for staff to learn and integrate new technologies and allowing as much flexibility as possible for individual preferences.

## Method

### Design and setting

The findings reported in this article are drawn from data collected during a wider study of technological change in UK general practice. Remote by Default 2 (RBD2) was a longitudinal ethnographic study of 12 general practices across England, Scotland, and Wales lasting for 28 months (September 2021 to December 2023) to follow the introduction (and in some cases subsequent withdrawal) of remote and digital services. We used an adapted researcher-in-residence model,^28^ whereby each case study practice had a dedicated researcher as their main contact, responsible for collecting all primary data from that site for the duration of the study,^29^ including interviews (individual and in small groups) and ethnographic observations.^30^ Focus groups were also used in two sites,^30^ due to their inclusion in a longitudinal sub-study that focused on workforce, which this article reports on.

Ethnographic observations took place during the first 6 weeks (for familiarisation) followed by every few months for the full 28 months, with the length of each visit being locally determined by practice preference, ranging from a few days at a time to 5 weeks of continuous participant observation. Researchers in residence also kept in touch by email in between visits. Interviews were conducted in person and remotely, and were ongoing for the full 28 months. The relevant workshop was held midway through the 28-month period, in January 2023.

As described in our protocol and baseline findings articles, the 12 practices were sampled to represent the full range of digital maturity from traditional (few digital services) to digital leaders (providing state-of-the-art digital services and supporting other practices).^29^^,^^31^ Additional contextual data were obtained from interviews with national stakeholders (mainly policymakers, plus some from industry and patient advocacy organisations), online multistakeholder workshops (one of which was centred on the workforce), and documents. Other sub-studies focusing on remote care-driven changes to safety,^13^ training requirements of staff,^18^ the needs and skills of reception and administrative staff,^24^ and equity in access and triage^21^^,^^22^ from RBD2 have been published.

The work was overseen by an independent advisory group including academics, policymakers, industry, clinicians, lay members, and a lay chair.

### Creating a subset of data for analysis on workforce issues

The entire study dataset was first loaded onto NVivo. To obtain a subset of practice-level data on the theme of workforce, we first asked RBD2 researchers in residence to highlight relevant interviews and observations from their fieldwork. We then searched the full RBD2 dataset (including ethnographic fieldnotes, interviews with practice staff and patients, national stakeholder interviews, and workshop transcripts) using preliminary codes, or keywords, identified through researchers’ inductive analyses during fieldwork, and subsequent group discussions thereon, such as ‘burnout’, ‘workload’, ‘stress’, ‘anxiety’, ‘time’, ‘flexible’, ‘change’, and ‘pressure’.

This provided us with the subset used for the analysis described here, consisting of extracts from fieldnotes in the 12 practices (approximately 1000 pages), interviews with 116 staff of various roles (approximately 3534 pages), and the transcript and chat archive of an online workshop on workforce including six break-out groups (80 pages total). Of our 116 interviewees, 66 were clinical (nursing staff of varying seniority/specialty, GPs of varying seniority, healthcare assistants, and trainees), 48 were in support roles (reception, administration, management, care coordination, and link workers), and two were mixed (phlebotomist–receptionists). All participants were anonymised using letters and numbers, and each practice has been given a pseudonym.

We also analysed relevant policy and policy-related documents including the Department of Health and Social Care’s *Plan for Digital Health and Social Care*^4^ and *Delivery Plan for Recovering Access to Primary Care*,^6^ the *NHS Long Term Workforce Plan*,^3^ the Health and Social Care Committee’s *The Future of General Practice*,^5^ and other grey literature, including reports from the British Medical Association^10^ and Care Quality Commission.^9^

### Data management, analysis, and developing a theoretical framework

As an initial step, we used thematic analysis guided by the above keywords to identify patterns in the data, combining close reading, broad thematic coding, discussion and reflection among the research team, refinement of themes, and selecting illustrative examples.^32^ However, we wanted to go beyond merely describing the impact (positive and negative) of changing work patterns on the general practice workforce. As such, we moved to an abductive analytical approach, searching for and testing relevant theories primarily in the sociological and organisational studies literature to better explain our data.^33^ This involved engaging with theories from information systems, psychology, internet studies, sociology, health services research, and organisational studies. We ultimately selected and combined three strands of theoretical work that fit our emerging analysis: technostress,^26^^,^^34^ organisational suffering,^27^ and the relational workplace;^24^ incorporating concepts from relational coordination^35^^,^^36^ and psychological safety.^37^ These are summarised in Box 1.

**Box 1.** Theoretical framework

For each concept/theory we indicate its (multi)disciplinary grounding. **Technostress: information systems, psychology, and internet studies** In 2008, Ragu-Nathan *et al* proposed five contributors to technostress in the workplace: overload, invasion, complexity, insecurity, and uncertainty.^26^ More recently, in 2021, Fischer *et al* added role stress (for example, job-related ambiguity), boredom (for example, where work is increasingly automated), control (for example, lack of autonomy in a work day) and (lack of) involvement in system design.^38^ Drawing on these and other sources, Umair *et al* refined a theory of technostress (geared towards their empirical work on online labour markets), which proposes that although technology can allow for greater flexibility and autonomy for workers, it also typically increases their stress, especially when work is intense and time constrained, when multitasking is required, and when workers feel penalised or coerced by factors beyond their control. If prolonged, technostress can lead to burnout, reduced productivity, loss of commitment, sickness absence, and high staff turnover.^34^ **Workplace suffering: organisation studies and bioethics** In 1998 Gill, citing Cassell’s definition of suffering as *‘the distress a person experiences when they perceive a threat to any aspect of their continued existence, whether physical, psychological, or social’*,^39^ showed that suffering in the workplace emerges from conflicts between staff members’ identities and their subjective experience of organisational control — both bureaucratic (to do with rules and roles) and normative (to do with shared values and organisational norms).^27^ When modes of control are compatible with their identities staff experience fulfilment, but when they are incoherent staff suffer. The multiple modes of control in an organisation may be more or less coherent with one another (for example, the rules and roles align closely with the organisation’s espoused values). A contemporary example of workplace suffering is the moral injury sustained by healthcare staff during the COVID-19 pandemic when treatment options and visiting time were strictly rationed and they could not provide the usual standards of care and comfort.^40^ There was both incompatibility (between the professional codes of conduct they internalised as part of their identities and the protocols they were required to follow) and incoherence (because these protocols also clashed with the espoused values of the healthcare organisations they were working in). To reduce their individual workplace suffering, workers may employ one of four strategies: blending (aligning aspects of their selfhood with modes of control), bridging (connecting selectively with those modes of control they find fulfilling), distancing (seeking to keep at bay certain contradictory modes of control through small acts of resistance), and separating (attempting to remove themselves from contradictory and incompatible modes of control through larger acts of resistance). **Relational workplace: sociology, organisation studies, and health services research** The term ‘relational workplace’ was applied by Dakin *et al* in 2023 to emphasise the importance of human relations for the effective workings of UK general practice teams, drawing on the work of Maben *et al*, which proposes that human action, interaction, and relationships enable the adoption, implementation, and long-term sustainability of innovations and improvements.^41^ Such relationships are seen as fundamental to the daily functioning of the practice. Furthermore, it is through positive relationships that the processes and routines of high-quality general practice are enacted, reproduced, and sustained over time. A relational approach to leadership and teams is an essential element of navigating continuous workplace change and crisis.^24^ This application of the relational workplace draws on Edmondson’s concept of psychological safety, defined as people’s perception that it is safe to take interpersonal risks (for example, by speaking up) in the workplace,^37^ and on elements of Gittell’s relational coordination theory, which foregrounds the importance of relationships and communication in achieving complex, interdependent work.^42^ As work tasks in health care become ever more fragmented and team based, the relational infrastructure of healthcare teams becomes critically important.^36^

A preliminary taxonomy of technostress, suffering, and relational behaviour in the context of contemporary general practice was produced and shared with staff participants, stakeholders, and our patient advisory group, and amended in response to their feedback.

We then brought our interim thematic analysis into close dialogue with our theoretical framework by organising extracts across all key themes and considering how each illustrated (or challenged) different kinds of technostress, the different ways in which suffering could be produced — and resisted — in the workplace, and different degrees of relationality. We actively sought examples of disconfirming data (where the framework did not fully explain the findings) to test and extend existing theory.

## Results

### Overview of findings

Our analysis gave a consistent impression that general practice staff were experiencing high levels of work stress, which they attributed variously to the intense and unremitting volume and pace of work, transformation of roles and ‘role creep’ (being asked to take on different tasks and responsibilities as required by new technologies), growing complexity and fragmentation of tasks (including increasing need to coordinate with others), the demands of new technologies, a perceived loss of control, and a feeling that standards of good professional practice were increasingly difficult to achieve. Broadly speaking, these findings align with those of previous studies.^17^^,^^20^^,^^21^^,^^24^^,^^43^

In the next sections, we expand on these findings, guided by our theoretical framework in Box 1.

### Technology’s strengths and stressors

Although all staff reported an increase in workload, our dataset contained examples of staff who had experienced the strengths technologies could afford general practice work, particularly improving autonomy and flexibility. For some practices, digital tools offered senior support staff and clinicians the opportunity to work remotely and have a better work–life balance. Some GPs not only did clinical consultations from home but also undertook remote training, research, and (in some cases) private-sector work, efficiently building a portfolio career around their home commitments. Others found that home-working risked blurring the boundary between work and home, with a negative impact on their work–life balance. The option of working from home was not equally available to all staff.

In some cases, digitalisation could speed up and automate work processes — for example, sending appointment information via email or text rather than postal letter, using a delayed messaging function to preschedule prompt messages for patients to book their follow-up appointments, or engaging patients and care coordinators in information gathering:

*‘Using different programs and software affected me a lot; now you can send a questionnaire to a patient and when you get a reply, the software does the recording and copying for you, and it gets coded into our system … it just made things a lot easier and quicker. Getting a response from patients via text, that was absolutely great, rather than rely on patients to call back or phoning them … care coordinators can chase up patients that haven’t responded … it just frees up our admin time.’*

(HQ, interview with GP and data-quality lead, Camp Street)

Support staff also recognised the benefits of digitalised systems for communicating with patients and managing patient requests, as described by a booking coordinator:

*‘*[The software] *is really good with communicating with patients with this triage system. In reception sometimes there’s a team of six or seven, sometimes there’s only two … When we get to book a patient, we don’t have to sit on the phone or call them three times, that takes too long. Now we get through 10 patients in the time we were taking calling one. So good text messaging software is essential for doing this job.’*

(BB, interview with booking coordinator, Newbrey)

However, these faster and more automated processes could also generate additional work elsewhere in the system. For example, digitalisation of discharge summary work had made this process near-effortless and near-instantaneous for secondary care staff, but this could mean that several versions with slight edits and corrections could be sent for one admission, creating de-duplication work for administrative staff in general practice, as outlined in this fieldnote excerpt from observations within an administrative team:

*‘I was with the admin team on the top floor. They are constantly working with different physical and digital documents, as well as all the software for communicating within the practice, and between the practice and hospitals.* [Practice manager] *has just been in to tell them he’s been getting complaints from the pharmacist and clinicians about having lots of duplicate letters attached to patients’ records.* [UC] *who is the team lead says that they can only cope with so much, and the duplication comes from the hospitals. Later, I asked* [UC] *to talk me through how receiving documents from the hospital works now that it’s all done via this document software. She tells me “The hospital fires over the discharge copy. If they’ve obviously forgotten to add something or they need to tell us something else, it duplicates the whole sheet with just that bit added on the bottom. Then maybe because of their changeover of shifts at the hospital, the next somebody comes in and resends the discharge letter. Sometimes I can get six documents that should be one. It makes me really angry, because I don’t have the time to sift through it. The doctors want it all dealt with ASAP. I get that, but I’m already running one person short, it’s pressure.”’*

(Fieldnotes from observations at Easton with UC, GP support supervisor)

We found many examples suggesting cognitive overload from (perceived) complexity of either the technology or the task, interruption (for example, from pop-up or on-screen messages), and other kinds of invasion, insecurity, or uncertainty, as well as role stress, monotony, and technologies that were unfit for purpose. Clinical staff discussed how pop-up messages could infringe on their professional autonomy, feeling they were delivering ‘care by committee’, and become an almost limitless source of additional (and largely unrecorded) work, as this GP outlined:

*‘“Screen Messages” is the bane of our lives because when there aren’t any slots available, everything goes onto the “Screen Messages”; it just can become never-ending. Lunchtime is when we start tackling them, which is sometimes a straightforward phone call to advise patients of how to take their medications, but sometimes more complex.’*

(CHK, interview with GP, Carleon)

Online tasks and remote consultations were often scheduled in greater volume than in-person work, owing to a presumption that digitalised work was faster and more efficient. This stretched workloads beyond what was feasible, as described by a nurse:

*‘The* [online clinical reviews] *has brought in a huge amount of extra work, they’re just there all the time. I think the expectation is we can do them quicker than if we were seeing them face-to-face.’*

(MS, interview with nurse manager, Fernleigh)

A receptionist reflected on the sustained upsurge in telephone calls following the mandate for remote-by-default access, and the associated rise in perceived abuse from patients:

*‘Before, we’d make 20 calls in the morning, but after COVID it went from 20 to 200. It’s still like that now, two and half years later… and a queue out the door. We lost loads of staff from it. People just left because they just couldn’t cope with the stress. Even yesterday, there was not one appointment on* [appointment management software]*, the patients think we’re lying to them. It’s a lot, the anxiety that we get knowing that we’re going to have no appointments to offer.’*

(QM7, interview with receptionist, Westerly)

These two factors combined to have a significant impact on the mental wellbeing of staff.

The increased accessibility offered by digital consultation request platforms often led to increased clinical workloads. Some technologically able patients used these platforms extensively (either to address previously unmet clinical need or, in some cases, for problems staff classified as trivial or self-limiting), thereby creating a form of supply-induced demand. These issues are outlined by a nurse and a GP in the following interview excerpts:

*‘*[Remote access] *captured so many patients that would never come for an asthma review … it’s grinding us down now, we’re a victim of the success of it.’*

(MS, interview with nursing manager, Fernleigh)

*‘I think, in a way, we’ve created a previously unmet need with these telephone consultations.’*

(CHK, interview with GP, Carleon)

Our findings affirmed that technology-supported work was more likely to lead to stress when the work was intense and time constrained, when multitasking was required, and when workers felt penalised or coerced by factors beyond their control. As we discuss later under ‘Distancing’, this could drive staff to move to part-time hours or leave practice.

### Technosuffering and fulfilment

Across observations and interviews, we encountered many examples of workplace suffering,^27^ which emerges when the organisation’s modes of control — either bureaucratic (to do with rules and procedures) or normative (to do with professional standards and moral codes) — are incompatible with staff identities or misaligned with one another. In our data, these modes of control included: task pathways dictated by technologies, protocols for how technologies should be used, and built-in features of technologies.

Examples of staff suffering stemming from misalignments between modes of control and staff identities, or between different conflicting modes of control, included: lack of availability of timely appointments because of supply and demand mismatch; patients struggling with remote access algorithms and waits; patients being triaged to a clinical team member who was perceived to be unsuited to their need; patients being offered a remote consultation when they wanted (or another staff member believed they needed) an in-person one; and patients who believed negative reporting of general practice in the media.

These misalignments were particularly evident during interactions with patients and when patients perceived the problem to reflect staff identities or standards (for example, a perception that staff *wanted* patients to be denied in-person appointments). Patients’ frustrations and fears about these scenarios would often be directed to the staff members. This elicited feelings of injustice, invisibility, and professional dissatisfaction in staff. These issues are illustrated in these two quotes:

*‘Patients can get aggressive and rude about a telephone wait. When you get sworn at down the phone on the first call. I just think, what am I doing here?’*

(SI, interview with patient services lead, Easton)

*‘I don’t think the media realises the impact of the way in which they portray GPs, that we aren’t seeing patients. Patients believe that more than they believe their own experience. What really upsets me is to see our secondary care colleagues believe that too, even though they’re also seeing people remotely. All of that builds up. You feel like you’re working harder now than you’ve ever worked. But public perception is that you’re not.’*

(TD1, interview with GP partner, Easton)

For many clinical staff, being ‘good’ in their professional capacities was intrinsically linked with in-person patient interactions, as illustrated by these two quotes:

*‘I much prefer seeing patients face-to-face. It’s fine for some, but for the annual reviews it’s much better bringing them in because we can see their inhaler techniques, or, for COPD* [chronic obstructive pulmonary disease] *patients if they’re losing weight you can see that, you can see their breathing … I do miss the patient contact as well; that’s why I’m a nurse. You get a better rapport in person.’*

(MS, interview with nursing manager, Fernleigh)

*‘I don’t feel like I’m as good a doctor when speaking on the phone. I think human contact is very key to delivering care to a patient. Those physical gestures of empathy and being human are harder to get across on the phone. It’s less job satisfaction for clinicians.’*

(NN, interview with GP partner, River Road)

Seeing their patients in this traditional way was a critical element in articulating their professional identity, engaging in professional practice, striving for professional standards of excellence, and achieving professional fulfilment. A requirement to engage with remote modalities that could misalign these articulations could impede professional fulfilment, causing a particular kind of dissatisfaction and suffering — which we refer to as technosuffering. Staff responded to these misalignments and injuries in various ways, which we have categorised using Gill’s terms: blending, bridging, distancing, and separating.^27^

#### Blending

Blending refers to how staff align aspects of their selfhood with modes of control to find partial fulfilment within new working constraints. In our data, staff could identify built-in features of technologies that were complementary to their existing working style. For example, this GP discussed the benefit of aligning their own slower consulting style with the built-in ambiguity of telephone consultation timings, enabling them to circumvent the 10-min limit on NHS GP appointments:

*‘I’m a slow consulter. I always have been … It feels less problematic keeping someone waiting if they’re expecting a phone call than if they’re sitting in a waiting room.’*

(OD4, interview with GP, Camp Street)

This GP was thereby using one mode of organisational control to overcome another. They felt that wait times experienced by remote patients were more acceptable than those experienced by in-person patients. They therefore used telephone waits as a buffer to enable them to engage in their ‘slow’ consulting style.

Some staff members would embody modes of control through becoming their workplace ‘self’, as discussed by this receptionist:

*‘When I go to work I’m getting on a stage; I’m doing an act. I’m quite a shy, reserved person. It feels like you have to perform because if not, it’s a lot more stressful.’*

(TR, interview with receptionist, Easton)

Reception staff described how they would develop a ‘thick skin’ and use a particular way of speaking (phrases and tone) when speaking to patients that often obscured their identities and reflected the modes of control in which they operated (for example, referring to practice policy, inflexible digital routes, and the limitations and non-clinical nature of their role). In some practices, this protective homogenisation was supported by shared training in telephony or ‘customer service’, and peer-to-peer training. Such intentional curtailing of staff individuality could risk a more transactional encounter between patient and service at the point of access.

#### Bridging

Incompatibilities could arise between the organisational modes of digitalised care and their professional guidelines or ‘mindlines’.^44^ To overcome these, staff used bridging: connecting selectively to modes of control they found fulfilling.^27^ This response was more common in roles with clearer professional guidelines, such as GPs and nurses. For example, we observed clinical staff selectively engaging with remote consulting depending on the specific patient or condition. In some cases, the staff member would bring patients in to confirm a provisional diagnosis or clarify symptoms discussed remotely. The fieldnotes here outline one GP’s experience learning to bridge between the limits of remote consulting and her professional mindlines:

*‘OD said that the 7 months spent working remotely had badly affected her mental health and her enjoyment of work. She worried about safety, triage, and quality when consulting remotely. But noted technology has offered more benefits than problems, especially in terms of information sharing. Eventually became happier with consulting remotely, but only with patients that she knows well. Patients could always elect to come in, and if she had doubts, she would request for in-person follow-ups.’*

(Fieldnotes from an informal discussion with OD, interview with GP, Camp Street)

This GP’s approach to blending remote and in-person consulting had restored enough professional satisfaction with her work that she could still find fulfilment.

Bridging requires additional articulation and hidden work from staff, which increases their cognitive load, risking mental harm and burnout. We identified examples of clinical and non-clinical staff becoming prone to isolation and overworking in attempts to meet their expectations for professionalism within these new modes of control, which could blur role boundaries, as highlighted by these receptionist’s and GP’s experiences:

*‘I come in out of my own time to clear the eConsult backlog because it gives me pure anxiety. I have to triage them, to decide how long they wait before the doctor calls them. That’s quite a lot of pressure. I’m so worried I’m gonna miss something.’*

(MY, interview with reception team lead, Westerly)

*‘I can’t get away from it* [working remotely]*. I felt like I had to sit there until I’d done all those consultations and I’d done all my results. Whereas when I’m in surgery, I get up, I walk around … it somehow feels less draining.’*

(OD4, interview with GP, Camp Street)

#### Distancing

Where modes of control conflicted with staff identities, they sometimes used distancing techniques — that is, small acts of resistance.^27^ In our data, these included workarounds for protocols or processes (digital and not) that functioned poorly, bending the rules, and learning to apply workplace policies creatively. Similar to bridging work, these acts of resistance could increase workload, as discussed by this GP who reflects on a ‘hidden consultation list’ (which had initially been a workaround) becoming integral to meeting patient demand:

*‘We have the Diary Book, where all consultations that basically can’t be fitted in end up. It’s like a third consultation list. It’s really awful … It’s normally meant to be just sick notes or results requests … It’s become this dumping ground for extra telephone calls.’*

(IK, interview with GP, Carleon)

Some distancing behaviour was a sympathetic response to the bridging work discussed above. Other distancing behaviours aimed to relocate staff in the ‘real’ world as a brief respite from the ever-present digital work. Some interviewees described temporarily physically removing themselves to escape the pressures:

*‘I just sit in my car. Just not to hear those phones going constantly.’*

(DX, interview with receptionist–phlebotomist, Rhian)

Whereas others sought some immediate support from their teams, as described in this excerpt from observations with a reception team:

*‘Someone is shouting down the phone at* [SI] *in reception, we can hear how loud the patient’s voice is from across the room.* [SI]*’s voice is wavering a bit but she is keeping quite level. She says, “Please stop raising your voice at me”, “I’m not here to be shouted at”, “I haven’t done anything”, “It wasn’t me you spoke to originally”. The call eventually ends,* [SI] *tells us that it was a pregnant patient who wanted them to call a hospital midwife for their blood results, which GPs can’t do.* [SI]*’s eyes are red, she’s upset.* [BU] *tells* [SI] *that she had to deal with the same patient; “They’re really difficult, it’s not on you. Why don’t you go have a cup of tea?”. They chat briefly about the interaction, which is enough to calm down* [SI]*. She then kept answering the incoming calls. Total exchange was under 5 minutes, but it was enough of a break/enough recognition to keep going.’*

(Fieldnotes from observations with the reception team at Easton)

#### Separating.

Occasionally, we encountered separating, when a staff member engaged in larger acts of resistance to remove themselves from modes of control. We saw examples of clinical and support staff requesting internal role changes, reduced hours, and GPs adopting a ‘portfolio’ approach to their careers to include multiple income streams, including private-sector work. This is discussed in the following quote, where a salaried GP had reduced their hours to take up private work that was not only better paid but also offered organisational modes of control that were better aligned to their identity and values:

*‘I do both NHS and private work. The computer system here is archaic. If the conditions were as good here, I wouldn’t work in private at all. If I could have the flexibility of private, the 20-minute appointments, no admin. Yeah, I’d just do NHS, I much prefer the concept of NHS.’*

(EO, interview with salaried GP, Easton)

We also saw evidence of all groups of staff choosing to leave their practices entirely. Cited reasons were the increased workload and more hostile working conditions (as outlined in a previous quote from QM7, a receptionist). Some left because they felt unsupported by their workplaces during the transition to remote care, and that associated privileges were offered unequally. Some reduced their hours because of the working conditions (as outlined in the quote from one of the GPs [EO] above) or fears of patient safety risks because of the high workload.

We also saw clinical staff who retired. One GP, for example, reflected that they would have retired sooner had they been forced to continue working remotely:

*‘Remote consulting was absolutely, totally unsatisfactory for professional and personal wellbeing … Like many GPs of my generation, I really think continuity is important … I think that I can manage my patients best, we can cut to the chase quickly. It’s much more rewarding. I value that relational aspect of general practice really highly. If I’d have stayed in remote consulting, I would’ve resigned because it was having such a bad effect on my mental health.’*

(OD4, interview with GP, Camp Street)

Many clinical staff discussed leaving but reasons for remaining related to a drive to care for patients, their passion for their work, and solidarity with their colleagues. Non-clinical staff were also retained through social bonds with colleagues and the reliability of the work income.

### Relational support and relational strain

Technostress and workplace suffering driven by the introduction of digitalised work processes had a powerful impact on staff relationships and their ability to coordinate and communicate effectively. In some places, these additional pressures elicited relational support between colleagues, where staff used their existing networks of relationships and communication pathways to curtail the impact of technostress and suffering. In others, they created relational strain, whereby the additional stressors frayed relationships and created friction in communication, compounding the impact of technostress and suffering, and having an impact on overall organisational efficiency. We arrived at our estimates of ‘how relational’ a given practice’s culture was through: 1) initial attention to how people behaved, interacted, and spoke with/about one another in our extended observations and interviews; and 2) through reviewing that data using the relational lenses outlined in our theoretical framework.

In some practices in our sample, interpersonal relationships were strong and positive with a history of effectively and harmoniously coordinating complex tasks. In such settings, staff appeared more able to maintain the social contact required to ensure continued coordination within these new modes. In the following quote, a GP discusses their frequent contact with reception throughout the day to ‘check in as we go’:

*‘It’s the volume, there’s too many things for one person to do. I go into reception quite a lot because I don’t think it’s healthy to stare at screens for the whole period that you’re* [the] *duty* [GP]*. And I think the duty session runs better if you’re in constant contact with* [reception] *as well while they’re adding requests to the task list. I don’t want to deal with everything* [on the task list] *at six o’clock, I like to check in as we go along.’*

(KE, interview with salaried GP, Easton)

This integration was more possible when staff had the time to learn and integrate these new technologies into established processes, understand where they fit into colleagues’ workloads, and how this had an impact on the integration of different roles’ tasks.

Where staff felt sufficiently psychologically safe to speak up, relational support was more evident. We observed clinical staff knocking on one another’s consulting doors to encourage them to come to team meetings or coffee breaks, ask their opinion on a case, vent frustrations, or deliver biscuits and tea to bolster morale. We also saw that relational strain could be confronted and off-set sufficiently to allow for more efficient practice function. The benefits of these supportive professional relationships were discussed by these focus group participants:

*‘I think we are incredibly lucky with our colleagues here. This is a place where you can come out, and somebody is always willing to listen if you’ve had a bad day or it’s been a busy shift. I’ve never had a duty* [doctor] *that’s not asked if I’m okay.’*

(MCY, advanced nurse practitioner, excerpt from focus group at Easton)

*‘I know when I just want to burst into tears, I can always say, “I’m going out* [for] *five minutes”, or I’ll just walk into someone’s room and say, I need to talk to someone. We’re very supportive of each other, which I imagine isn’t the case for every healthcare setting.’*

(SI, patient services lead, excerpt from focus group at Easton)

Practices in our sample with weaker interpersonal relationships tended to have more evidence of relational strain. This was particularly the case, for example, in practices that were very hierarchical, where teams were socially and physically separated, or when ‘cliques’ had developed within/between teams. Relational strain was also more evident when staff perceived inequities in the benefits that digitalisation could afford, or when workloads were unequally distributed. These issues were made starkly clear in the observations described in the following fieldnotes, where two receptionists working in practice formed an in-group that excluded those working remotely, which strained communication and created a tense working environment when those with work-from-home privileges came into the practice:

*‘It’s 9 am. Reception is sparse today. There are supposed to be five of them, but one has COVID, one has a doctor’s appointment and is supposed to work from home before, and the practice manager is working from home. The reception team lead and the receptionist are the only ones in, and they are angry, suggesting those on WFH* [work from home] *aren’t working. “I don’t mean to moan but it’s a bit much for two people to handle mate. It’s the same people over and over again going off to work at home, and it’s the people in charge who get paid twice as much as us. I’m meant to be out front* [on the desk] *but there’s too many e-consultations.”* (QM7) *“Is* [receptionist] *even working from home? It ain’t fair they get to! Is she answering calls? The prescription folder’s empty she should be logging in and doing calls.”* (MY) *“Yeah but she’ll say she has other work to do, now* [practice manager] *has gone off* [to WFH] *it’s hard to make sure* [WFH receptionist] *does work. I don’t mean to sound like I’m moaning but it’s hard going. It ain’t fun, but I don’t wanna complain too much cause I don’t want them to think we can’t cope.”* (QM7) *They continue to make comments throughout the morning about the two people working from home. That afternoon, the* [WFH receptionist] *with a doctor’s appointment comes in, and it is very tense. The two who have been here all day are frosty with her and whisper between themselves.’*

(Fieldnotes from observations at Westerly)

## Discussion

### Summary

This longitudinal study of UK general practice has shown that remote and digital working has offered opportunities for improved process efficiency, patient communication, and information gathering. However, in many cases, integrating and adapting to these new technologies has exacerbated already high levels of workplace stress through additional ‘technostress’,^26^ leading to unique forms of technology-induced workplace suffering.^27^ Staff have developed strategies to mitigate technostress and technosuffering, and sometimes achieve fulfilment through a range of blending, bridging, distancing, and separating tactics. More extreme distancing and separating behaviours tended to reflect staff coming closer to their breaking points, being driven to work in a manner that was increasingly misaligned with their professional identities, as indicated by some choosing to leave the practice or move to part time. Some teams demonstrated relational support for one another to acknowledge and reduce suffering. Critical to this was a psychologically safe and relational working culture. Where relational support was lacking, technostress deepened relational strain among teams, compounding suffering.

Our findings demonstrate that digitalised processes and care delivery in primary care offer some benefits (such as increased efficiency) in some tasks and processes in primary care. However, these benefits may come with a human cost, which is often experienced unequally across internal hierarchies. Digital technologies increase the complexity and fragmentation of working in general practice, introducing new challenges and interpersonal strain when conducting interprofessional work within and between clinical and support staff teams. We saw challenges to professional satisfaction, where staff felt they could not perform their ideal of a ‘good’ doctor/nurse/receptionist, requiring them to either change roles or learn to adapt. Indeed, we found instances where staff that experienced suffering in response to these new organisational controls had adapted their working style to maintain or re-attain professional fulfilment, which was critical in preventing total resistance to system change (and, therefore, critical to retain them in the workforce).

Engaging with theory from other disciplines, most significantly organisation studies, has significantly enriched our analysis. The lenses of technostress,^34^ organisational suffering,^27^ and relationality in the workplace enabled us to see the staff-level benefits and consequences of the digitalisation of tasks and routines in UK primary care in a new way.^24^^,^^37^ Gill’s proposed strategies for overcoming suffering were particularly useful in helping us to identify how staff were able to individually overcome technostress and suffering driven by digitalisation, such that they could continue to find fulfilment in their work (or endure it). These concepts supported us in identifying where particular staff members’ hard limits were for such endurance and at what point they would separate entirely. Our chosen relational concepts helped us to see the collective impact that technostress and technosuffering had on relationships within practice teams; whether that was to elicit strain, support, or both.

This theoretical framing has also provided indications as to *why* staff endure these forms of stress and suffering. Alongside practical and financial drivers for employment, staff outlined an unchanging commitment to patients, to their practice(s), and to their colleagues as significant factors in retaining their positions. We intend to develop deeper understanding of these relational components for enduring such turbulent working conditions in future research.

### Strengths and limitations

We drew on detailed ethnographic fieldnotes and rich narrative interviews with staff and focus groups from a diverse sample of practices, to document their changing experiences during a crucial period in the history of UK general practice from mid-2021 to end 2023. The researcher-in-residence model allowed us to develop good working relationships with participating practices and capture both the positive and negative impact of the changes. However, our data on the experiences of certain groups of staff with vulnerabilities (for example, those with protected characteristics) were more limited. This is an area that could merit further research.

We would also note that there were external factors playing out during some of our data-collection period (for example, when COVID-19 pandemic restrictions were still in place). Hence, our data need to be interpreted accordingly. While our sample included sites and participants from practices in England (*n* = 8), Wales (*n* = 2), and Scotland (*n* = 2), we were not able to recruit Northern Irish practices, which should be noted when interpreting these findings. We also note that policy documentation reviewed for this study was focused on England and Wales. Our findings may not be transferable to other settings (for example, secondary care and non-UK).

### Comparison with existing literature

Workloads in UK general practice have continued to rise beyond pre-pandemic levels, following a brief initial dip during the pandemic’s first few months.^45^^–^48 This new normal of higher workloads is compounded by a growing shortfall between the supply and demand of available primary care staff with more staff reducing their hours, leaving the service, risking burnout, and facing abuse at work.^45^^,^^47^^,^^49^^–^52 Current conditions enhance the funding, workforce, and capacity frictions faced by primary care pre-pandemic.^52^^,^^53^ Our article makes a novel contribution by outlining where digitalisation has contributed to these increased workloads, and the human cost of the work required to achieve both digitalisation and good patient care. We also expand on the experiences of support staff in general practice, a significantly underresearched group,^24^^,^^54^ and place them on equal footing with their clinical colleagues.

Beyond the UK context, it is notable that a study of the introduction of video consultations in Denmark uncovered similarly complex interactions between the introduction of that technology, the organisational contexts, and staff (GP’s) identities and values.^55^ Another found that intrinsically motivated GPs were more likely to experience burnout when exposed to external change.^56^ This resembles our finding that staff who experienced too much contradiction between digital modes of control and their professional identities would engage in distancing or separating behaviour. Direct comparative research on the digitalisation of general practice between the UK and other countries would be a fruitful area of further research.

In terms of theoretical contributions, we expand the application of technostress beyond other digital settings like the gig economy into digital primary care delivery for the first time.^26^^,^^34^ We also present, to the best of our knowledge, the first instance of applying Gill’s conceptualisation of workplace suffering to digital work in UK general practice, to identify its unique modes of control — and how staff resist them to maintain fulfilment.^27^ Finally, we have extended the concept of the ‘relational workplace’ to identify mechanisms of ‘relational strain’ and ‘relational support’.^24^

### Implications for research and practice

Our findings highlight that theory-informed approaches to research and policy add significant value in enabling us to abstract beyond the boundaries of the empirical setting, to gain insight into the after-effects of introducing complex change into a complex sociotechnical system. Such approaches also afford us the language to describe and communicate the issues under study and to learn from similar settings. In this setting in particular, Gill’s theory of workplace suffering has provided us with a lens by which we can appreciate the complex responses that changes to workplace routines can have on the heterogeneous workforce of general practice, and thus may add nuance to debates on, and responses to, the system-wide workforce shortages and retention crisis.

At the level of the practice, there should be recognition of the technostress experienced by some clinical and non-clinical staff because of digitalised work processes and care delivery. Space must be given to practices to determine locally what technologies and change to processes work best for their staff, and how to design protocols around their use to minimise the impact of workforce stress and improve job satisfaction and retention. Practice leaders and managers should continue to work with each clinical and support staff member to ensure that, as far as possible, the balance between new and digitalised processes/modes of consulting is adaptable to each person, such that they are professionally satisfying, manageable, and safe. Practices should clearly communicate changes to existing role boundaries and responsibilities, and acknowledge that any changes are processual, requiring periods of adjustments and adaptations to be achieved. Where possible, staff members in digitally heavy roles should be offered variety in their workflows and have wide team engagement built into their working days to support the endurance of relational structures. Staff must be given the time to learn and integrate new technologies into established processes, understand where they fit into colleagues’ workloads, and how this has an impact on the integration of different roles’ tasks. This could be well supported by relationally informed training programmes to develop and strengthen individual practice’s preconditioning for change and crisis management, as has been successful in secondary care teams.^57^^,^^58^

Policy must be directly informed by the reality of the workplace in which it seeks to make change. Without such real-world grounding, policy can be hard to implement for practices and individuals, creating more friction for staff. For example, although policies to support flexible working exist, as set out in the NHS ‘flexible working principles’^59^ and the ‘flexibility by default’ commitment in the NHS People Plan,^60^ their application in practice is not widespread.

In conclusion, our findings highlight the unique challenges that the integration of digital technologies in UK general practice has created for staff across the primary care workforce. We have gone beyond description, with a novel application of Gill and Ragu-Nathan *et al*’s concepts to reconceptualise these challenges to integrating new digital technologies as ‘technostress’ driving new forms of workplace suffering.^26^^,^^27^ This suffering changes what it means to work in general practice across all roles, both in terms of day-to-day functions or tasks, as well as the professional identity and morality associated with these roles. Although our application of the ‘relational workplace’ enabled us to identify how certain teams had been able to support one another through these forms of technostress and technosuffering, we also learned that not all teams had the capacity to support one another through these difficulties.^24^ In a sector that faces its most severe workforce crisis in history, it is critical that the suffering experienced by staff be addressed and rectified as much as possible. Without mechanisms to protect from technostress, and rebuild the social structures for relational support, the caustic nature of control-driven workplace suffering will continue to erode general practice teams. This will further weaken the healthcare system, affecting the health of patients and staff.
